# Solving the Puzzle: Molecular Research in Inflammatory Bowel Diseases

**DOI:** 10.3390/ijms241713389

**Published:** 2023-08-29

**Authors:** Susanne M. Krug

**Affiliations:** Clinical Physiology/Nutritional Medicine, Charité—Universitätsmedizin Berlin, 12203 Berlin, Germany; susanne.m.krug@charite.de

Inflammatory bowel disease (IBD) encompasses chronic idiopathic relapsing and remitting gastrointestinal autoimmune diseases characterized by chronic inflammatory disorders of complex etiology, posing clinical challenges due to their often therapy-refractory nature. The primary disorders within the IBD classification are ulcerative colitis (UC) and Crohn’s disease (CD), sharing similarities but exhibiting distinct differences, sometimes making their discrimination challenging.

A prominent feature of IBD is the inflammation of the intestinal mucosa, characterized by the robust and persistent infiltration of immune cells and compromised intestinal barrier integrity, leading to the phenomenon known as “leaky gut.” The inflammation can manifest acutely or chronically relapsing and can increase in severity over time, thereby causing life-long morbidities and reduced quality of life for affected individuals, underscoring the need for a deeper comprehension of the molecular contributors to disease pathogenesis and progression.

Despite extensive research, the etiology of IBD is still not fully understood, and so far, existing treatments are inadequate to effect a complete cure. The disease’s multifactorial nature implicates genetic, environmental, infectious, and immunologic factors as key contributors. The dysregulation of both transcellular and paracellular intestinal barriers, along with the activation of mucosal immune responses, either as a consequence or a trigger, play pivotal roles in the pathological manifestations.

Recent developments in IBD research are broadening our overall understanding of the disease enabling the discovery of novel molecular predictive indicators and facilitating the creation of cutting-edge therapeutic approaches. This Special Issue presents a comprehensive compilation of diverse facets that contribute to the advancement of solving the puzzle of IBD.

In general, impaired homeostasis is known to be critical for the development of IBD. A multitude of factors and components are involved in the physiological maintenance of homeostasis and are found to be affected or to be an effector in IBD. For example, muscarinic acetylcholine receptors (mAChRs) play a significant role in maintaining intestinal epithelial homeostasis, and their activation is essential for the maintenance and reinforcement of epithelial function. Non-neuronal acetylcholine systems are also recognized to be involved in mAChR activation in epithelial cells. A review in this Special Issue summarizes recent advances in research on mAChRs and non-neuronal acetylcholine systems as potential targets for therapy in treating IBD [1].

Shifts in the oxidative status of the intestinal epithelium may not only affect the epithelium but also gut-microbiota homeostasis. Hypoxia-inducible factor-1α (HIF-1α) is one of the central players for that. The lysate from the probiotic formulation SLAB51 was found to increase HIF-1α levels in human intestinal epithelium under normoxic conditions, leading to higher glycolytic metabolism and protection from lipopolysaccharide (LPS)-induced inflammatory response. The probiotic treatment stabilized HIF-1α via the activation of the PI3K/AKT pathway, resulting in an inhibition of NF-κB, nitric oxide synthase 2 (NOS2), and an increase in the IL-1β triggered via LPS treatment [2].

Another key player for mucosal inflammation has been discussed to be the paracaspase MALT1, particularly in the context of IBD. MALT1’s proteolytic activity was shown to be involved in inhibiting ferroptosis and promoting STAT3 signaling, which is essential for regulating immune and inflammatory responses as well as mucosal healing. These mechanisms might be used for the identification of novel therapeutic targets for the treatment not only of IBD but also other inflammatory diseases [3].

Immune cells are one main group of important players in the development and progression of inflammatory processes in IBD. In this Special Issue, for example, the role of polymorphonuclear neutrophils (PMNs) and their significance in promoting colonic pathobiology IBD and colon cancer, which can develop from IBD, is discussed. Special focus was laid on intracellular lipid droplets (LDs) and transcription factor FOXO3 in PMNs. The presence of FOXO3-deficient PMNs could be associated with increased transmigratory activity, differentially expressed genes linked to metabolism, inflammation, and tumorigenesis, and predicts colon cancer invasion and poor survival [4].

Food antigen-specific effector T-cells that may respond to barrier disruption and antigen in patients with celiac disease (CeD) were also found in comparable levels in active CD patients. The frequency and phenotype of nutritional antigen-specific T-cells in these two patient groups correlated with the presence of small intestinal inflammation, indicating that active inflammation in the small intestine plays a crucial role in the development of peripheral food antigen-specific T-cell responses in CD as well as in CeD and could be a key factor [5].

A new and innovative methodical approach to identifying immune cells is a label-free optical technology, which utilizes autofluorescence using NADH and FAD signals. These can be utilized to classify and characterize different immune cell subtypes and their activation states in the context of IBD. This study demonstrates the value of autofluorescence as a tool for identifying innate and adaptive immune cells, determining their relative amounts, and distinguishing their functional states, which could lead to a label-free clinical classification of IBD in the future [6].

Besides the intestinal immune system, another major factor in IBD is the epithelial barrier. The claudin family of tight junction proteins is a crucial component of intestinal barriers, and their altered expression and localization in IBD may lead to intestinal barrier dysfunction and worsen immune hyperactivity and disease. While claudins are known to control the passage of ions and water between cells, emerging evidence suggests additional non-canonical functions during mucosal homeostasis and healing after injury, leaving the question open whether claudins play adaptive or pathological roles in IBD responses. Analyzing current research, it is hypothesized that claudins’ versatility might come at the cost of specialized mastery, potentially leading to conflicts between maintaining a robust claudin barrier and facilitating tissue repair, thereby exposing vulnerabilities in the barrier’s integrity and compromising overall tissue healing during IBD [7].

To study the epithelial barrier in joint context with immune cells Le and colleagues developed an advanced in vitro inflammation-triggered triple-culture model involving the human intestinal epithelial cell line Caco-2, mucus-producing goblet cell line HT29-MTX-E12, and macrophages of different origin. This model demonstrated characteristics of a “leaky gut” upon an inflammatory stimulus and could be valuable for screening and evaluating therapeutic drugs for the treatment of IBD, including potential IL-23 inhibitors that were analyzed in that model [8].

In IBD, there is significant activation and remodeling of mucosal micro-vessels. The role of the gut vasculature in inducing and persisting mucosal inflammation is increasingly recognized, where endothelial cell activation and angiogenesis are thought to promote inflammation. On the other hand, the vascular barrier may offer protection against bacterial translocation and sepsis, which indicates that other barriers besides the intestinal epithelial one are of importance in IBD. One review of this Special Issue focuses on examining the different phenotypical changes observed in the microvascular endothelium during IBD and presents potential vessel-specific targeted therapy options for the treatment of IBD [9].

Another review focuses on diseases other than IBD, which affect the intestine. These may give insights of general importance and could link symptoms and treatments of more than one disease. Behçet’s disease (BD) is a chronic and recurrent systemic vasculitis involving almost all organs and tissues. Intestinal BD may have severe gastrointestinal complications and share similarities with classical IBD, particularly active CD. The review highlights the dysregulation of immune function as one of the main pathogenes in both, intestinal BD and IBD. It emphasizes the potential of biological agents, particularly anti-tumor necrosis factor agents, as effective treatment options for patients with refractory intestinal BD, a therapy that is also employed in IBD [10].

IBD is often associated with the development of colorectal cancer. Despite extensive studies of IBD pathogenesis, the molecular mechanism of how IBD is promoting tumorigenesis is not yet fully understood. Through a comprehensive bioinformatics analysis of transcriptomics data from mouse models of acute colitis and colitis-associated cancer (CAC), a set of key genes involved in the regulation of colitis and CAC was identified. These genes, particularly matrix metalloproteinases (MMPs), can potentially serve as novel prognostic markers and therapeutic targets for controlling IBD and IBD-associated colorectal neoplasia. Additionally, a translational bridge connecting these genes with the pathogenesis of UC, CD, and CAC was established [11].

Taken together, this Special Issue brings together new research and reviews covering various aspects of IBD. It includes studies by joining functional, genetic, and molecular research as well as innovative methods. By doing so, it helps piece together multiple aspects of the complex puzzle of understanding IBD.
